# Cognizance of Molecular Methods for the Generation of Mutagenic Phage Display Antibody Libraries for Affinity Maturation

**DOI:** 10.3390/ijms20081861

**Published:** 2019-04-15

**Authors:** Chia Chiu Lim, Yee Siew Choong, Theam Soon Lim

**Affiliations:** 1 Institute for Research in Molecular Medicine, Universiti Sains Malaysia, Penang 11800, Malaysia; jenniferlim294@gmail.com (C.C.L.); yeesiew@usm.my (Y.S.C.); 2 Analytical Biochemistry Research Centre, Universiti Sains Malaysia, Penang 11800, Malaysia

**Keywords:** human monoclonal antibodies, phage display, combinatorial libraries, affinity maturation

## Abstract

Antibodies leverage on their unique architecture to bind with an array of antigens. The strength of interaction has a direct relation to the affinity of the antibodies towards the antigen. *In vivo* affinity maturation is performed through multiple rounds of somatic hypermutation and selection in the germinal centre. This unique process involves intricate sequence rearrangements at the gene level via molecular mechanisms. The emergence of *in vitro* display technologies, mainly phage display and recombinant DNA technology, has helped revolutionize the way antibody improvements are being carried out in the laboratory. The adaptation of molecular approaches *in vitro* to replicate the *in vivo* processes has allowed for improvements in the way recombinant antibodies are designed and tuned. Combinatorial libraries, consisting of a myriad of possible antibodies, are capable of replicating the diversity of the natural human antibody repertoire. The isolation of target-specific antibodies with specific affinity characteristics can also be accomplished through modification of stringent protocols. Despite the ability to screen and select for high-affinity binders, some ‘fine tuning’ may be required to enhance antibody binding in terms of its affinity. This review will provide a brief account of phage display technology used for antibody generation followed by a summary of different combinatorial library characteristics. The review will focus on available strategies, which include molecular approaches, next generation sequencing, and *in silico* approaches used for antibody affinity maturation in both therapeutic and diagnostic applications.

## 1. Introduction

In the past decade, monoclonal antibodies (mAbs) have become the prevailing class of biologics in the biomedical and biopharmaceutical arena. This is evident from the increasing number of pharmaceutical companies offering a range of antibody-associated products and the growing number of antibodies undergoing clinical studies [1,2]. As of December 2017, a total of 57 mAbs (for cancer and non-cancer indications) have been approved for the U.S. and E.U. pharmaceutical markets [3]. The global sales revenue for mAb products was approximately U.S. $75 billion in 2013, surpassing the U.S. $98 billion set in December 2017 and is projected to reach at least U.S. $137–200 billion in 2022 [3,4]. The continuous market growth is largely a consequence of the rapid approval rate and high demand for mAb products, which are considered to have lower safety issues than other therapeutic products. Of all the mAb products on the market, Humira has recorded nearly U.S. $11 billion of sales, which was the highest recorded for a therapeutic product [4]. However, most mAb products are expensive due to their high manufacturing costs, being difficult to manufacture, and requiring higher dosages [5]. For example, a course of anti-CTLA-4 treatment would cost U.S. $12,000 (Bristol-Myers Squibb), which is relatively expensive [6]. The production of monoclonal antibodies has also been significantly improved for better process yields (scalability and capacity) and a reduction in manufacturing costs. In doing so, such products can penetrate cost-sensitive markets to benefit more people [3,4]. The accelerating development and commercialization of mAbs (against existing and extended indications) will contribute to the breadth of the therapeutics market and allow them to dominate as the major class of biologics in the coming years.

The instinctive binding ability of mAbs against a target antigen with great specificity and affinity has propelled mAbs to their role in the diagnostic and therapeutic field [7,8,9]. The specificity of an antibody is defined by the complementarity determining regions (CDR) of the variable domains against a specific target. It is programmable *in vivo* by two important molecular mechanisms, namely V(D)J recombination [10,11,12] and somatic hypermutation (SHM) [13,14]. These events greatly influence the final sequence diversity of an antibody repertoire [15]. A greater understanding of protein architecture and the development of new molecular techniques have allowed for easy and rapid insertion of foreign DNA material into the genome of a filamentous phage [16]. This technology has become the basis of antibody phage display for the development of recombinant mAbs against a plethora of targets [1]. The introduction of antibody phage display has allowed laboratories to generate human antibodies *in vitro* without the need for host immunization as opposed to the conventional hybridoma technology [17]. The technological developments have also allowed for novel derivatives of native antibodies to be introduced. The presentation of different binding entities, such as single chain variable fragment (scFv), antigen-binding fragment (Fab), variable fragment (Fv), and its derivatives, has allowed for additional flexibility and a broader application of antibodies [18]. The choice of antibody format used in phage display is largely subjected to the size constraints associated with phage display presentation [19,20]. The construction of combinatorial antibody phage libraries utilizing B-cell mRNA from human peripheral lymphocytes, either from healthy or diseased donors, yields stochastic naïve and immunized libraries, respectively [21]. The scFv is the most common format used for phage display due to the size and availability of both the heavy and light variable chains (V_H_/V_L_) for a collective binding effect [22]. The V_H_ and V_L_ domains are joined by a flexible protease-resistant glycine–serine linker (GlySer) to form a functional scFv [23]. The combinatorial mixture of genes forms the basis of a fully functional antibody repertoire in the combinatorial phage library [1]. The combinatorial antibody library functions to mimic the natural antibody repertoire through the presentation of unique antibodies by billions of phage particles [24].

## 2. Phage Display Technology: Harnessing Novel Antibodies

Phage display technology was first introduced by George P. Smith [16]. It enables proteins or peptides of interest to be presented on the surface of a filamentous phage via genetic fusion of the proteins or peptides to phage coat proteins. Since then, this method has been widely adopted in molecular display approaches. The underlying feature of phage display is the utilization of the surface coat proteins of a bacteriophage to provide the physical linkage of the genotype and phenotype [25]. The basis of phage display technology can be attributed to the understanding of the biology of M13 bacteriophage that enables technological improvements to be made. At present, phage display technology comprises of wild phage display [26,27,28] and hybrid display systems [28,29,30,31,32] that differ in the vector types used [33,34].

Filamentous bacteriophages from the genus *Inovirus* have a filament-like structure, consisting of a circular, single-stranded DNA (ssDNA) genome [35]. Filamentous phages are capable of infecting gram-negative bacteria, including *Escherichia*, *Salmonella*, *Xanthomonas*, *Vibrio*, *Thermus*, and *Neisseria* [36]. Among the filamentous phages that infect *Escherichia coli*, the most well-characterized phages are the F pilus conjugative specific bacteriophages (Ff phages). These phages infect *E. coli* strains that bear an F pilus conjugative plasmid. M13, f1, and fd phages are categorized as Ff phages due to their highly similar genome sequence (98.5%) and their mode of infection via the presence of an F pilus [37,38]. The ssDNA genome of an Ff phage is approximately 6400 bp in length and encodes for 11 different genes. These genes are divided into structural proteins (genes III, VI, VII, VIII, IX) and functional proteins that are required for phage replication and assembly (genes I, II, IV, V, X, XI). On top of that, the phage genome also contains an ori site (origin of replication) that is responsible for the production of (+) and (−) DNA strands. Another site, known as the ‘packaging signal’, is responsible for initiating phage assembly. The Ff virion appears to be 900 nm in length with a diameter of 6.5 nm. The ssDNA genome of Ff virion is fully enclosed in a flexible, cylindrical compartment made up of 2700 molecules of the major envelope proteins, pVIII. The asymmetric ends of the Ff phages are comprised of five copies of the minor coat proteins pIII and pVI on one end, and the other end carries pVII and pIX [28,39,40]. The role and function of the envelope proteins have been reviewed extensively by Mai-Prochnow et al. [35] and Rakonjac et al. [36]. One distinguishing characteristic of Ff phages is their ability to replicate without killing the host bacteria. Once the host bacterium is infected, the Ff phage replicates as an extrachromosomal element without integrating itself into the host chromosome, allowing for the host bacteria to shed viral particles continuously. Hence, M13 and related Ff phages are preferred to the lytic phages, such as T4 and T7 [21].

To date, hybrid phage display systems have been preferred to wild phage display systems. The wild phage display platform (Type 3 and Type 8 systems) allows for multivalent display of the recombinant fusion protein [39]. The phage vector carries all the essential genes required for infection, virion replication, assembly, and budding, as well as the recombinant fusion gene (fused to the N-terminal of either gIII or gVIII) [40]. The presentation limitations of wild phage display systems are such that the size of the displayed polypeptides will greatly affect the efficiency of viral packaging and infectivity [16,28,30,41]. This issue can be resolved with the use of hybrid display systems. One of the hybrid display systems adopts a phagemid-based system that enables the plasmid that carries the phage coat protein fusion to be packaged as phage particles. Such a hybrid display system consists of Type 3 + 3 and Type 8 + 8 systems based on the choice of coat proteins [18,40]. The phagemid vector (4.6 kb) is a plasmid that encodes several key elements, including the bacterial and phage origins of replication, a leader sequence, multiple cloning sites, an antibiotic resistance gene, a phage coat protein gene (gIII or gVIII), and a weak promoter (*lac*Z) [29]. Notably, a phagemid vector alone is not capable of producing infective phage particles. A helper phage, such as M13KO7 or VCSM13, is indispensable to provide the necessary genes encoding all the wild-type coat proteins essential for phage replication, packaging, and assembly [42]. The helper phage is a modified Ff phage that has a defective packaging signal (M13 intergenic region); therefore, the replication and packaging is less efficient compared to a phagemid vector that carries the wild-type M13 intergenic region. To produce a fusion protein, *E. coli* harbouring the phagemid vector is superinfected with the helper phage. Initially, the phagemid vector replicates in *E. coli* as dsDNA. Upon coinfection with helper phage, ssDNA and the phage coat proteins are produced, thus initiating the packaging and release of mature phage particles [40].

**Figure 1:**
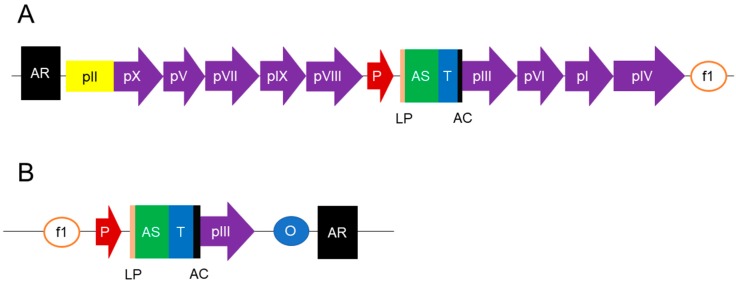
Construction of antibody libraries can be done either using a phage or phagemid vector. An antibody sequence (AS) is located between a leader sequence (LP) and a pIII coding sequence (pIII). Both vectors consist of a promoter (P), an antibiotic resistance (AR) gene for selection, a tag (T) for detection or purification, and an f1 origin (f1 ori) for replication of single-stranded DNA and generation of antibody-displayed phage particles. Phagemid vectors have in addition a plasmid origin (plasmid ori) for propagation in *Escherichia coli*. An amber stop codon (AC) is essential for both phage and phagemid vectors and located in between the antibody and pIII coding sequences. Phage vectors will have the genes of the other coat proteins (pX, pV, pVII, pIX, pVIII, pVI, pI, and pIV), whereas phagemid vectors only have the pIII gene.

Unlike phage vectors, the phagemid vectors have a smaller size and are relatively easily cloned, thus improving the transformation efficiencies and enabling larger libraries to be generated [34,40]. An amber codon (TAG) is commonly available in phagemid vectors, and is inserted between the antibody gene fragment and the coat protein gene. This allows for soluble expression of antibody fragments devoid of the phage coat protein in non-suppressor *E. coli* strains, such as HB2151. Also, it allows for the presentation of antibody fusions to the phage coat protein in suppressor *E. coli* strains, such as TG1 or XL1-Blue [29]. The hybrid phage display enables monovalent display of the fusion protein because the resulting phage particles often carry only a single copy of the fusion protein. In addition, only 10% of the phage particles or less will present the fusion protein. Type 3 + 3 and 8 + 8 systems enable the selection of higher-affinity ligands as the selected ligands possess true intrinsic affinity for their cognate molecules [39], while the high avidity effect of multivalent display may be derived from numerous low-affinity interactions [43]. Recently, a modified helper phage, known as a hyperphage, was introduced to improve the display level of fusion antibodies [44]. It has no functional gIII, but exhibits the wild-type pIII phenotype. This allows the hyperphage to remain infective, while the sole source of pIII needed during phage assembly is supplied by phagemid-encoded pIII fusion [42]. Hence, the display level of the fusion protein is multivalent, akin to a wild phage display platform. This display method is able to lower background noise during the selection process, whereby interference of empty phages without the fusion protein is reduced. On top of that, the avidity effect increases the chance of acquiring positive binders [34]. Currently, many types of helper phage are available, and they have been reviewed by Chasteen et al. [45]. A schematic diagram of a phage vector and a phagemid vector is given in Figure 1.

**Figure 2:**
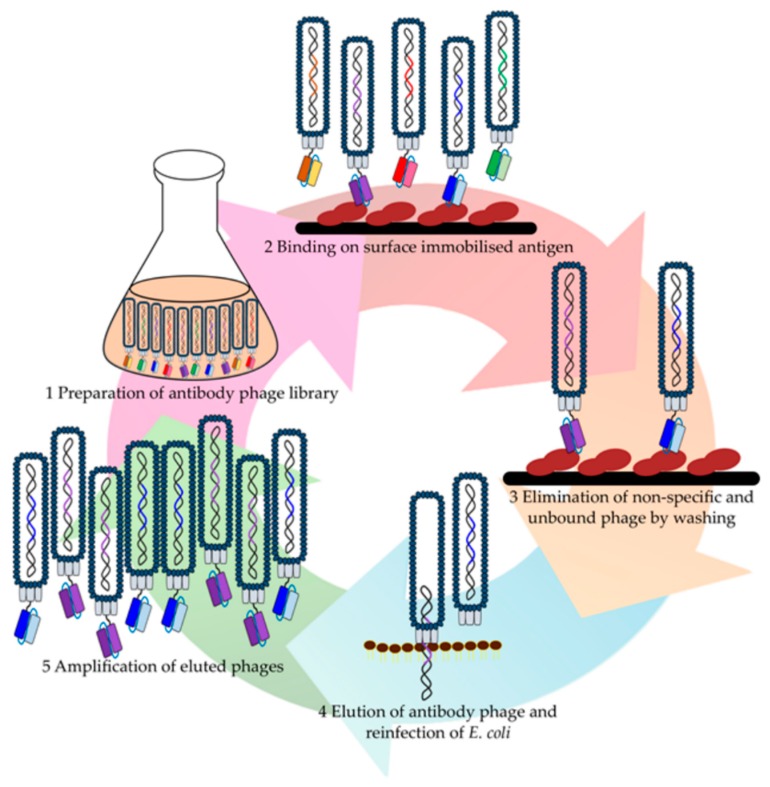
The biopanning process utilizing an antibody phage library against various target antigens. The process encompasses a few steps, including (1) preparation and (2) purification of the antibody phage library, and (3) repetitive binding, washing, (4) elution, rescue, and (5) amplification of the bound phages. The process is usually repeated three to five times to obtain an enrichment population.

Knowledge of the function and role of each coat protein in the replication process allows for the manipulation of phage particles to present larger proteins, such as antibodies [46,47]. This presentation process allows for the isolation of the phenotype and rapid identification of the subsequent genotype in a single process [28,39,48]. The enrichment of the clones is carried out via continuous rounds of a panning process. The panning process allows for affinity-based enrichment of clones via a process that encompasses repetitive steps of binding, washing, rescue, and re-infection [42,49,50] (Figure 2). The process starts with the introduction of the phage population to the target molecules for binding. Then, a wash step is introduced to remove any unspecific binders from the target. This step is normally varied between rounds to increase the level of stringency. At this stage, nonspecific and weak binders are liberated from the target molecules. The leftover bound phage particles are then rescued prior to another round of panning. After several rounds of panning, usually between three and four rounds, an enriched population is obtained and can be identified [39,51]. The enriched pools from each round of panning (polyclonal pool) can be evaluated via enzyme-linked immunosorbent assay (ELISA) to determine the level of enrichment. The phage from the outperforming round (normally from the last biopanning round or otherwise intermediate rounds) will then be subjected to monoclonal ELISA to screen for monoclonal antibodies. Thereafter, the selected monoclonal antibodies will be identified by DNA sequencing to retrieve the cognate antibody sequence [17,51]. Biopanning is normally done by utilizing a collection of antibody-presenting phage particles known as antibody libraries.

## 3. Combinatorial Antibody Phage Library

An antibody molecule consists of two binding domains: the variable domain of the heavy chain (HC) and light chain (LC) that preferentially or collectively contribute to the binding affinity of the antibody towards the target antigen [52]. An antibody phage library is constructed in a manner that allows for a single antibody pool to represent a myriad of antibody gene sequences. Hence, in order to replicate the natural antibody repertoire offered by the immune system, diverse antibody repertoires can be achieved by randomly combining both the HC and LC repertoires during antibody library construction [53]. The availability of human germline V_H_, V_κ_, and V_λ_ gene segment sequences, as well as D- and J-segment sequences, has allowed for better amplification coverage of the V-gene repertoire with a set of specific primers [54,55]. The source of antibody repertoire used for library preparation has a great influence on the types of antibody libraries being generated. It may be derived from different hosts other than humans [56,57,58,59], and the different immune responses in healthy and disease states [60] will have a profound impact on the diversity of the antibody repertoire [61]. In light of this, antibody phage libraries can be classified into two main types with respect to the source of V_H_ and V_L_ gene segments used during library construction. Natural antibody phage libraries are comprised of V-genes acquired from immune [21,62,63] and non-immune donors [64,65,66]. Synthetic antibody phage libraries consist of V-genes that are designed to be either partly or completely synthesized *in vitro* [67,68,69].

### 3.1. Naïve Antibody Libraries

A naïve antibody library is a collection of immunoglobulins derived from circulating B-cells found in primary and secondary lymphoid tissues (bone marrow, spleen, and tonsils) or peripheral blood [70]. The rearranged V-genes of the IgM isotype from healthy or non-immunized donors are used primarily to generate naïve antibody libraries with a large library size (up to 10^11^) [63,71]. Other than humans, naïve repertoires could also be harvested from animal sources that give rise to different origins and formats. Under natural circumstances, an individual is expected to possess at least 10^8^ antibody-producing B-cell clones [53]. A single pot library produced from multiple donors is capable of generating antibodies against almost all types of antigens, such as peptides, toxins, and self-antigens. This is due to the clonal diversity of B-cells that generates a diverse population of antibodies capable of targeting a wide range of foreign antigens [71]. The broad application of naïve libraries has allowed them to be used to develop antibodies against targets relevant to cancer [72], autoimmune diseases [73,74], infectious diseases [17,75], and other diseases for either diagnostic or therapeutic applications. Generally, the naïve repertoire is polyreactive because most of the B-cell clones are non-activated and is comparable to those during primary immune responses [40].

This obvious shortcoming is sometimes overlooked in favor of the benefits provided by naïve libraries. This includes active immunization of human donors and ethical issues that can be avoided due to the direct usage of V-gene repertoires from naïve libraries. Both non-immunogenic and toxic antigens can be targeted with no adverse side effects. Also, mAbs with a broader range of binding affinities are obtained against one single antigen at one go. The most important fact of a naïve library is the ability of such a naïve repertoire to target multiple antigens with no prior exposure [18]. Despite the ability to screen for multiple antigens, a naïve library comes with huge drawbacks; the antibodies obtained are often lower in affinity compared to those from immune libraries and have a higher possibility of cross-reacting [42,71]. Nevertheless, this issue is normally circumvented by constructing antibody libraries with a larger size and diversity. Larger libraries could possess sub-nanomolar affinities, whereas smaller libraries could deliver up to micro- to lower-nanomolar range affinities [42,76]. Alternatively, antibody affinities can be improved via *in vitro* affinity maturation processes post-identification [77].

### 3.2. Immune Antibody Libraries

To generate an immune antibody library, IgG mRNA is obtained from immune donors, such as disease-infected patients. Ideal samples that can be collected from patients are those that are either undergoing acute infection or in the recovery stage, as well as patients that have recovered from a particular infection or disease [42]. The choice of sample is subjected to the mechanism of the disease and downstream application of the library [72]. One unique feature of immune antibody libraries is that the sample materials are obtained from activated B-cells, in which the B-cells are activated during the antigen encounter and subsequently undergo affinity maturation processes. Thus, the predisposition of antibody clones to recognize certain antigens is pronounced and allows for the isolation of high-affinity binders specific to the target antigen [40].

Immune libraries are useful tools to study the humoral responses against different diseases and stages of infections [78]. Unlike naïve libraries, the presence of the biased V-genes in the antibody repertoire post-exposure to an antigen gives rise to a high number of antigen-specific antibodies. On top of that, antibody clones that have undergone affinity maturation processes (i.e., somatic hypermutation and clonal selection and expansion) will be in high-copy numbers, therefore increasing the likelihood of enriching high-affinity antibody clones [18]. As a result, the size of an immune library need not be as huge as that of a naïve library [79]. Recently, Moon et al. reported the isolation of antibodies against multiple non-immunizing antigens, suggesting that immune libraries also have the capacity to contain a large amount of unimmunized clones [80]. This provides the possibility that large immune libraries will appear that are as useful as the naïve library. Therefore, it is conceivable that a diverse enough immune library may also work much like a naïve library to target other antigens due to the presence of unimmunized clones. This reflects the extended breadth of protection provided by the B-cell memory in the immune system to an individual [81].

Nonetheless, immune libraries are associated with several drawbacks. In contrast to naïve libraries, immune libraries are not well-suited to target a large panel of antigens, especially against self-antigens. This is because the immune system has developed immunological tolerance towards self-antigens. However, this is not the case for immune libraries derived from autoimmune diseases, where the targets are mainly self-antigens [18]. Another profound limitation of immune libraries is the generation of immune repertoires utilizing human donors. Generally, donor samples from humans are limited to disease-infected patients only and are difficult to obtain [42]. The biased nature of the immune repertoire is mainly useful against the antigen of the specific disease; therefore, new libraries are needed when targeting antigens from different diseases as immune libraries are generally generated with modest diversities [55,82]. There are also ethical issues related to sample attainment, as it is not feasible to apply active immunization to humans and animal donors with deadly antigens (i.e., toxins or immunosuppressives) for isolating their corresponding mAbs [18].

### 3.3. Semi-Synthetic and Synthetic Antibody Libraries

As opposed to naïve and immune libraries that utilize naturally occurring sequences, semi-synthetic libraries consist of partially natural sequences mixed with chemically synthesized sequences. This would allow for a natural framework to be maintained and the diversity of the antibody library to be designed [77]. This is normally done utilizing gene synthesis approaches to provide a random collection of sequences that will be used as the CDR [83,84]. This allows for an artificial diversity to be generated as a consequence of unifying the natural and synthetic sequences. The advantage to designing a semi-synthetic antibody library is the pre-determination of framework sequences to be used [68,85]. This will aid in the downstream antibody panning success and application. Specific frameworks can be used to ensure greater success as particular frameworks, such as V_H_3-23 (DP47) for improved (thermodynamic) stability and reduced aggregation [86,87,88], V_λ_1-47 (DPL3) [83,87], V_λ_3-19 (DPL16) [89,90], and V_κ_3-20 (DPK22) [91,92] for higher expression and improved stability. Overall, several combinations of V_H_/V_L_, such as V_H_3_κ_3, V_H_3_κ_1, V_H_1_κ_3, and V_H_5_κ_3, have been reported to have excellent expression yields and thermostability [93]. This point is also a noteworthy point for the development of fully synthetic antibody libraries.

The main difference between a semi-synthetic and a synthetic library is that the entire antibody sequence used in a synthetic library is chemically derived. Unlike other antibody libraries, a synthetic antibody library is an artificially created repertoire, often by *in vitro* reconstruction of V-genes via CDR randomization [69,94]. This library is a true single-pot library as compared to a naïve library [95] because it is free from natural biases and redundancies arising from evolutionary influence and can target many different kinds of antigens [96]. Synthetic libraries are also useful for targeting non-immunogenic, toxic, and self-antigens [77,97]. The construction of a synthetic library is fundamentally based on *de novo* synthesis and the antibody diversity is designed *in silico* [98]. Synthetic libraries can be further categorized according to the types of framework used, the origin and design of sequence diversity within CDR, and the library generation methods [71]. The large diversity in a synthetic library is afforded by predefined framework designs and the degree of diversification of the CDR [99,100]. The *in silico* design of the synthetic antibody repertoire is basically generated from a collection of bioinformatic analyses using existing experimental data, including antibody epitopes, antigen–antibody interactions, affinity maturation designs, variable gene segment recombination, and structural predictions on variable regions. These studies have provided valuable information on amino acid predominance and variabilities in CDR regions [101]. Table 1 summarizes the different kinds of antibody libraries that are available together with their critical characteristics.

## 4. *In Vitro* Affinity Maturation Strategies

Recombinant mAbs obtained from combinatorial libraries are often diverse and comparable to those generated by the immune system [71]. Despite the fact that combinatorial libraries are able to produce antibodies with improved affinities and specificities, it is not always possible to gain antibodies with those desirable features [120]. Affinities and specificities of mAbs can hugely affect the antibody activities, including antigen binding, pharmacokinetics, effector functions, and the efficacy and safety profile [121]. In light of this, antibody optimization can be conducted by employing affinity maturation approaches based on mimicking the *in vivo* processes at the *in vitro* level to yield improved antibodies [77]. Note that genome editing technologies are not discussed in this review as our focus is mainly on *in vitro* methods that do not include methods that offer gene manipulation at the *in vivo* level, i.e., gene modifications within live organisms [122].

### 4.1. Random Mutagenesis

Traditionally, the directed evolution of an antibody library employs random mutagenesis as a means to introduce variation in antibody sequences. Generally, the mutations are introduced randomly at a fixed region along the antibody sequence [123]. This is usually targeted at the three CDR, with CDR3 being the main target [124,125]. This approach is considered to be more straightforward than the rational design approach as modifications can be done without the need for any structural information [126]. Several different approaches are commonly used to achieve this level of mutagenesis in an antibody library context for the selection of affinity-matured antibodies.

#### 4.1.1. Error-Prone Polymerase Chain Reaction (PCR)

Error-prone PCR is an established universal method for random mutagenesis that leverages the natural error rate of a low-fidelity DNA polymerase, i.e., *Taq* polymerase [127,128]. The workflow for mAb identification abides by the conventional panning strategy. Upon selection of the ideal mAb clone for affinity maturation, error-prone PCR amplification is then performed to introduce mutations at the CDR to generate a miniature library of mutants [129,130]. This is then followed up with another round of selection to obtain improved candidates. By manipulating DNA amplification conditions, ideal mismatches can be created due to the nature of the polymerase that lacks 3′-5′ proofreading ability. A commercially available *Taq* polymerase, Mutazyme (Agilent Technologies), was engineered for error-prone PCR to reduce its mutational bias for prohibitive selection preference on certain nucleotides during amplification [131,132]. Meanwhile, MutaGen™ is an *in vitro* random mutagenesis approach that utilizes low-fidelity human DNA polymerase, known as mutases [133]. Mondon et al. employed this method to extensively diversify the frameworks and CDR of the human antibody variable domains [134]. Alternatively, gene modification can be achieved by performing isothermal rolling circle amplification (RCA) under error-prone conditions, producing ssDNA with multiple tandem repeats during amplification. This method is able to generate a random mutant library with a wild-type sequence as a template [135,136]. Error-prone PCR has been applied to numerous studies to enhance antibody affinities, such as hapten-specific antibody fragments (an increase in affinity by 4.5-fold) [137]. Additionally, coupling with DNA shuffling was also done to improve anti-fluorescein scFv to 100 fM [138].

Error-prone PCR is not site-directed and can lead to mutations occurring at random sites along the entire sequence. This can result in the introduction of additional interacting residues at the interaction sites as well as the framework regions. Therefore, the three-dimensional structure of the antibody contact regions may be disrupted. As it is random, it may also introduce mutations that could enhance the interaction points or even the structural stability of the antibody [139]. Several other parameters can be adjusted to alter the fidelity of the polymerase: (1) the concentration of *Taq* DNA polymerase, (2) the concentration of the bivalent cations Mn^2+^ and Mg^2+^, (3) the concentration of deoxyribonucleoside triphosphates (dNTPs), (4) the elongation time, and (5) the number of PCR amplification cycles [130,140].

Error-prone PCR is only able to generate limited base substitutions and is well-suited for determining amino acid residues that are associated with antigen function, affinity, and specificity [141]. Therefore, extensive amounts of transitions can be achieved in the resulting library, involving A→G and T→C, leading to a possible amplification bias due to high GC content. However, an ideal random mutagenesis method sets out to equally substitute all nucleotides and to achieve maximum amino acid change when the three consecutive nucleotides are substituted. In an ideal condition, all four transitions (AT→GC and GC→AT) and eight transversions (AT→TA, AT→CG, GC→CG, and GC→TA) would be expected to occur at equal amounts and at a desired probability with no insertion or deletion being detected [142]. Therefore, a Poisson distribution of mutation is expected to occur. Even so, it has been shown that error-prone PCR produces a broader non-Poisson distribution of mutations [143]. It was also reported that having a high mutation rate may result in more unique sequences, but few actually retain their function and vice versa. Therefore, an optimum mutation rate would provide the best outcome with a balance between clonal uniqueness and functionality. Other than phage display, error-prone PCR is versatile enough to be adopted for use in other display platforms, such as yeast [144], ribosome [145], and mRNA [146] displays.

#### 4.1.2. Chain Recombination

Gene modification and diversification can be achieved by means of gene recombination. Such rearrangements are highly recommended for obtaining mutational combinations that are beyond those naturally available. A ‘mix-and-match’ mechanism is provided by chain shuffling, enabling a repertoire of one chain (either a heavy or light chain) to be paired with the partner chain (this chain is kept constant), giving rise to a secondary repertoire [71]. Such domain shuffling is capable of mimicking *in vivo* SHM, resulting in affinity improvements from a universal effect by the swapped variable domains [147]. This does not produce specific refinements, much like when variations are introduced to the CDR. Even so, chain shuffling of antibody fragments has been used for the identification of improved antibodies against haptens [148,149,150], proteins [151], carbohydrates [152], and receptors [153].

Another variant of gene recombination uses *in vitro* homologous recombination, known as DNA shuffling, by random fragmentation of a pool of closely related gene sequences and reassembly of the fragments by PCR [154]. Such gene recombination leads to template switching to produce a myriad of new sequences, providing extended sequence diversity to the gene pool [155]. A universal approach provided by Meyer et al. [156] initially creates double-stranded breaks at the regions of interest using DNaseI, followed by denaturation of strands and reannealing at homologous regions. The hybridized fragments will then serve as templates that are subjected to repeated rounds of extension, denaturation, and annealing to form new diverse sequences. DNA shuffling is touted to be more superior to error-prone PCR and oligonucleotide-directed mutagenesis. This is because DNA shuffling does not suffer from the possibility of introducing neutral or non-essential mutations from repeated rounds of mutagenesis. DNA shuffling can overcome this issue by backcrossing with high amounts of a wild-type DNA sequence, i.e., the shuffling process is performed with an excess parental sequence [157]. Although the error-prone PCR approach promotes high mutational rates, the sequence space is actually very much untapped [156] and the observed substitutions of improved clones are often distant from the binding site, suggesting that poorly understood mechanisms are involved in functional improvement [158]. In light of this, further DNA shuffling with hypermutated clones is beneficial to attain functional improvements that cannot be achieved by error-prone PCR [143].

### 4.2. Site-Specific Mutagenesis

Site-specific mutagenesis is an *in vitro* gene modification approach that involves a defined gene locus or specific regions of DNA sequences to study the candidate at sequence, structural, and functional levels [159]. To perform site-specific mutagenesis, structural data is needed, and they can be derived from previous random mutagenesis studies where important mutational and tunable positions were identified [160]. On the contrary, site-saturation mutagenesis [161] substitutes targeted sites with all possible amino acid residues, elucidating the importance of a specific amino acid residue towards antibody function through focused mutagenesis [162,163]. This is suitable for engineering antibody stability by studying the effect of different amino acids at potentially strategic positions along the antibody structure and can be performed in accordance with the readily available structural data and homology modeling [164,165,166]. In addition, a comprehensive search of a mutational sequence space can be done by using degenerate oligonucleotide primers [167,168]. An in-depth review has been provided by Ruff et al. [163] and Siloto et al. [169].

#### 4.2.1. Enzyme-Based Mutagenesis

Site-specific mutagenesis can be conducted using several methods. The readily available restriction nucleases and DNA ligases facilitate the incorporation of mutagenic sequences into templates for recombinant DNA constructs [170]. Due to the rapid and simple synthesis of oligonucleotides, oligonucleotide-mediated mutagenesis is widely employed to assist with site-specific mutation by providing internal mismatches that direct point mutations or multiple mutations to the target DNA sequence [171,172]. For example, a mutagenic primer is designed to complement the ssDNA template, subsequently elongated by a Klenow fragment of DNA polymerase I, and is finally ligated by T4 DNA ligase. The heteroduplex DNA is then transfected into a competent *E. coli*, giving rise to a mixture of transformants with either mutant or wild-type DNA [159].

Kunkel mutagenesis utilizes a circular, ssDNA with the incorporation of uracil as a template to synthesize a double-stranded DNA (dsDNA) product in accordance with an oligonucleotide primer that introduces a mutation [173]. The dsDNA is then transfected into the *E. coli* dut^−^/ung^−^ strain, and the bacterial repair mechanism cleaves the uracilated parent strand, leaving the recombinant clones to propagate. This approach is particularly useful for phage display involving an M13 bacteriophage that consists of an ssDNA genome [174,175,176]. A technical refinement of Kunkel mutagenesis was performed to produce mutant peptide phage libraries of 10^11^ clones by relying on the amber stop codon TAG in the coding region of pIII of bacteriophage M13. The oligonucleotides with the designated mutations will anneal to the ssDNA M13 genome, rendering the randomized region to form a heteroduplex with TAG. Enzymatic extension along the oligonucleotides will then result in a closed circular dsDNA, and mutants can then be selected using non-suppressor *E. coli* strains [177].

The previously mentioned site-directed mutagenesis method described the use of a ssDNA template with a labor-intensive process that includes numerous subcloning and ssDNA rescue steps [159,174]. Recently, several commercial kits have become available to provide solutions for site-specific mutagenesis using dsDNA as a template and with the assistance of mutagenic primers. The QuikChange™ system [178,179] amplifies the entire plasmid with high-fidelity polymerase using a pair of complementary oligonucleotides (forward and reverse) that are designed with the desired mutations. Upon amplification, the parental molecule is removed by *Dpn*I endonuclease [175]. A variety of QuikChange™ (Stratagene Inc.) derivatives has been summarized by Tee and Wong [142]. The GeneTailor™ system (Invitrogen) is very much similar to QuikChange™, except that the former requires methylated DNA as a template. The GeneEditor™ system (Promega Corp.) uses antibiotic resistance to identify cloning vectors that confer ampicillin resistance [180,181].

Another PCR-driven site-specific mutagenesis method is overlap extension PCR (OE-PCR). This technique is rather simple, employing four oligonucleotides to generate modified genes in just a few steps [181]. The target gene segment is amplified from a DNA template, utilizing two flanking master primers and two internal primers. The internal primers are designed to consist the desired mutation and overlapping sequences. Initially, the target gene is subjected to PCR amplification using the internal primer sets, producing two gene fragments that share some overlapping sequences at their 3′ ends. The double-stranded duplexes are then denatured and re-annealed, generating two heteroduplexes with mutations at each strand. The overlapping ends of each heteroduplex are then extended by DNA polymerase. A second PCR is then performed using two flanking master genes to amplify the entire modified gene [182,183,184]. This method is commonly employed to conduct massive single amino acid mutagenesis in parallel with microarray-based DNA synthesis technology. The convergence of both strategies allows for a powerful tool to screen for mutants in a high-throughput manner [185].

Apart from conventional directed evolution that relies on digestion and ligation, other enzymes with different functions can be utilized for mutagenesis. Lim et al. reported the ability to carry out DNA shuffling using an ssDNA template and lambda exonuclease [186]. In nature, lambda exonuclease assists in DNA recombination of viral DNA. It is an exonuclease that degrades dsDNA progressively from 5′→3′, especially the phosphorylated chain of a duplex DNA, resulting in mononucleotides and ssDNA [187,188,189]. Initially, the targeted DNA fragments of homologous genes are each amplified by one 5′-phosphorylated primer and a normal (non-phosphorylated) primer. The lambda exonuclease selectively degrades the 5′-phosphorylated DNA strands and the ssDNA templates are obtained. The ssDNA templates are then subjected to overlapping PCR with a Klenow fragment for dsDNA assembly. Site-specific mutagenesis of an antibody gene can be performed using degenerate oligonucleotides at a specific region [186]. However, the major limitation of this strategy is the lack of codon specificity for directed evolution, and crossover sites have to be predetermined [142].

A recent method for antibody chain shuffling was developed by Lai et al. that is devoid of restriction endonucleases and exonucleases. The approach takes advantage of the hybridization kinetics of DNA as well as the sequence specificity of DNA hybridization. The method involves a two-step methodology to create a chain-shuffled mutant repertoire, involving the preparation of gene cassettes followed by cassette hybridization by conventional PCR amplification. The gene cassettes were initially prepared by PCR amplification, followed by vector assembly with the desired gene combinations through hybridization using solely DNA polymerase at optimized conditions. The chain-shuffling method yielded a comparable outcome with respect to the conventional restriction digestion and ligation method [190].

Alternatively, *in vitro* somatic hypermutation rendered by the activation-induced cytidine deaminase (AID) enzyme has also been applied for diversifying an antibody repertoire very much like how it mediates the *in vivo* somatic hypermutation process [191]. The AID enzyme belongs to the apolipoprotein B mRNA editing catalytic polypeptide-like (APOBEC) family of cytidine deaminases that catalyze the *in vitro* deamination of cytidine residues to uridine residues on ssDNA, resulting in thymine residues after replication events [192]. Generally, the cytidine residues are targeted at the mutational hotspot motifs RGYW and AGY (where R = A/G, Y = C/T and W = A/T), and these are the preferred motifs for *in vivo* somatic hypermutation as well [193,194]. Apart from ssDNA targets, the AID enzyme is also able to deaminate dsDNA *in vitro* in a transcriptional-oriented manner; hence, it is useful for gene diversification [195,196]. AID-mediated mutagenesis serves as a strategy to enhance antibody sequence diversity by introducing various point mutations, including single-amino-acid substitutions or indels (insertions, deletions) and it is particularly meaningful to impose on antibody CDR regions in a localized manner. Furthermore, some complicated mutations, such as nucleotide transversions and duplications, could be performed using this approach as well as clonal expansion for the directed evolution of antibodies [197]. An analysis of the amino acid diversity of both a germline and a mature antibody showed a decrease in the number of germline hotspots in high-affinity antibodies. The outcomes suggested that *in vivo* affinity maturation was responsible for the somatic mutation at the hotspots [198]. Therefore, it would be sufficient to focus *in vitro* randomization at the short CDR regions by mimicking the natural *in vivo* SHM event [199]. The permissive mutational hotspots are embedded in the amino acids that are directly or indirectly involved in antibody–antigen interactions [198], thus serving as suitable candidates for mutagenesis by AID enzymes or other trans-acting hypermutation factors [200].

Alanine-scanning mutagenesis is widely employed for a systematic substitution of amino acid residues with alanine for the identification and characterization of functional epitopes and catalytic residues. Replacement with alanine eliminates the influence of all the side chain atoms beyond the beta-carbon. Hence, this method is also useful to study the role of side chain functional groups at specific positions of a protein [201]. It has been reported that both computational and experimental alanine-scanning mutagenesis was applied to identify the most permissive sites in CDR regions of sdAbs specific to alpha-synuclein prior to mutagenesis [202]. Due to the laborious workflow of conventional single-site alanine-scanning mutagenesis, methods have been developed for multiple alanine substitutions in a high-throughput manner [203]. Combinatorial alanine scanning has also been presented as a high-throughput approach to analyze multiple positions, utilizing split-pool synthesis of degenerate oligonucleotides (one pool for the alanine codon and another for the wild-type codon) [204]. Shotgun alanine-scanning mutagenesis, which renders tetranomial substitutions (of wild-type, alanine, and two other amino acids), has been applied with phage display to map the complete antigen-binding sites of an anti-ErbB2 [205]. On top of that, Robin et al. demonstrated the first quantitative statistical analysis on multiple antigen–antibody complexes via computational alanine scanning to understand antigen–antibody binding and derive characteristic rules that may be helpful for antibody design and library generation [206]. Of note, an alanine stretch mutagenesis instead of single-site or multiple-site alanine substitutions was also performed and found to be a feasible approach for epitope mapping as well [207].

Iterative saturation mutagenesis (ISM) is a novel site-specific mutagenesis approach with an iterative feature. The strategy includes saturation mutagenesis at three (maximum) sites, resulting in a total of 12 mutant libraries. This method is able to demonstrate the role of additives and/or cooperative effects of mutants in the libraries [208]. The B-Factor Iterative Test (B-FIT) focuses on protein scaffold stability and is able to guide improvements, such as to thermostability. The B-factor value (also known as temperature factors) indicates the scaffold mobility of a protein and is calculated from X-ray crystallographic protein (antibody) structures. The B-FIT approach first requires B-FITTER to select important regions (hotspots for mutagenesis) based on the determined B-factors. A high B-factor value indicates highly flexible regions of the protein scaffold while applying ISM at such regions with the aim to increase the rigidity and improve the thermostability [160]. A recent report employed B-factor analysis to analyze a human peripheral blood antibody repertoire, typically the V_H_-CDR3 loop, and revealed that affinity maturation does not necessarily cause rigidification [209].

Cassette mutagenesis is another variant of site-directed mutagenesis that entails restriction enzyme digestion and ligation to introduce mutagenic sequences [210]. Different from error-prone PCR, which focuses on relatively short regions of a gene, this approach is convenient for larger targeted DNA sequences (up to 100 bp) flanked by the restriction endonuclease cleavage sites that do not excise elsewhere in the plasmid [211,212]. The desired DNA fragment can be cleaved by the complementary restriction enzymes and can be replaced with ssDNA or dsDNA that carries the desired mutations. With the aid of oligonucleotides with designated mutations, the target gene is amplified with a series of oligonucleotides forming ‘megaprimers’. Subsequent annealing into the plasmid and transfection into *E. coli* gives rise to a mutant pool [213]. This approach is very much similar to that used for the construction of the HuCAL library. With respect to large-scale mutagenesis, this method can be optimized by using spiked synthetic oligonucleotides that carry different mutations, allowing randomization of one or several cassettes in any given region of the target gene [132,214]. The conventional cassette mutagenesis relies on Kunkel mutagenesis, which is very time consuming, while a recent improved Kunkel methodology, named PFunkel mutagenesis [215], enables mutagenesis to be performed in just one day [160]. PFunkel mutagenesis was used to prepare mutant antibody libraries for epitope mapping of antibodies against tumour necrosis factor (TNF), pertussis toxin, and the cancer target TROP2 [216]. The main constraint of site-specific mutagenesis is the time-consuming task of primer design. Rational design is important to introduce desired mutations at precise positions. Tools, such as AAscan, PCRdesign, and MutantChecker, are now available to assist researchers during the mutagenesis process [217].

#### 4.2.2. Chemical-Based Mutagenesis

Chemical-based mutations are mainly focused on the application of chemical methods or chemically derived mutations to generate antibody mutants. Saturation mutagenesis (SM) seeks to achieve mutation at a maximal capacity by examining substitutions of a given residue against all possible 19 amino acids and can be done on multiple residues; however, the handling is physically impractical [218]. The method accommodates degenerate primers that encode for a mixture of sequences at the targeted codons for randomization [219]. NNN degenerate primers are widely employed for mutant library generation, as the degeneracy gives rise to all possible 64 variant combinations. This includes three stop codons that cause difficulties during screening and increase the probability of enriching non-functional clones due to the random introduction of termination codons [160]. Hence, reduced codon sets were introduced to lower the codon redundancy and frequency of terminations, including NNK, NNS, and NNB codons (where N = A/C/G/T, K = G/T, S = C/G, and B = C/G/T) that also encode 20 amino acids [219]. Despite being able to reduce the probability of prematurely truncated variants, these degenerate primers are expensive to synthesize, and it is impossible to use a single degenerate primer to reduce codon redundancy while providing all 20 amino acids [220,221].

Several strategies have been implemented to circumvent these limitations, ultimately to reduce the library size without compromising the functional variants. One of the methods involved in the synthesis of redundancy-free mutagenic primers is the use of mono-, di-, or tri-nucleotides phosphoramidite solutions (see below) [218]. A sophisticated randomization procedure using ‘MAX’ has superior characteristics over randomization with degeneracy. ‘MAX’ results in equal probabilities of all 20 amino acids without encoding termination codons, and has been reported to successfully eliminate library redundancy [221]. The ‘MAX’ randomization selects a collection of codons (MAX codons) that is favorable for expression of each amino acid in *E. coli* and generates randomization cassettes from a single template oligonucleotide via selectional hybridization. The selection strand is amplified (distinguishable by primers) to produce random cassettes that are enzyme-digested for cloning, giving rise to multiple combinatorial randomized genes [222]. This non-degenerate saturation mutagenesis strategy was further improved to extend the saturation coverage to multiple contiguous codons, dubbed ‘ProxiMAX’ randomization. ProxiMAX involves the ligation of a blunt-end dsDNA donor bearing the intended MAX codons at terminal ends to a blunt-end dsDNA acceptor. The resulting strands are amplified, purified, quantified, and combined (at desired ratios) during the next randomization cycles. The restriction endonuclease *Mly*I is used to eliminate the DNA donor strand throughout the randomization process, leaving the randomized sequence as the acceptor strand for subsequent ligation cycle. The frequency and diversity of codons are well-controlled by recycling different sets of donors. ProxiMAX randomization has been applied to antibody engineering by saturating 11 consecutive codons within the V_H_-CDR3, with the resulting distribution of the desired amino acids being satisfactory [223]. Recently, Frigotto et al. integrated the ProxiMAX methodology into an automated setup named ProxiMAX Colibra^™^. The setup was able to perform high-throughput codon randomization using hexamer nucleotides in a single reaction that is equivalent to two cycles of conventional ProxiMAX (where hexamers are premixed and ligated as a pool then subjected to PCR amplification, purification, and lastly digestion by *Mly*I) [224].

Solid-phase combinatorial gene synthesis is also able to generate a highly diverse library with no biases (see below). A more cost-effective strategy can be executed for routine SM experiments that uses normal primers to achieve near-zero or zero redundancy [218]. The ‘Tang’ scheme demonstrates the use of a mixture of four primers, NDT, VMA, ATG, and TGG (where D = A/G/T, V = A/C/G, M = A/C) at each randomized position with a molar ratio at 12:6:1:1, to create zero probability of premature termination codons and equal distribution for each of the 20 amino acids (theoretically a 1/20 distribution) [220]. The ‘22c-trick’ scheme uses only three primers, NDT, VHG, and TGG (where H = A/C/T) at a 12:9:1 molar ratio, and results in zero probability of premature termination codons and near uniform amino acid distribution: 2/22 for Leu and Val, and 1/22 for each of the remaining 18 amino acids [221]. There are other different primer mixing strategies that can be employed to create these ‘smart-intelligent small libraries’ even with the differences in their underlying concepts [225,226,227]. However, the selection of an ideal strategy is very much dependent on the outcome of the library to be achieved based on size and quality as well as the costs involved [218,228].

In order to carry out effective site-specific mutation, the exact position of critical residues in the antibody sequence has to first be identified. This would require a complementary analysis using *in silico* methods to predict critical residues for mutation either based on pattern recognition or by structural information [229]. Computational software, such as B-FITTER [208], Patch-Finder [230,231,232], and Rate4Site [233], has been introduced to assist in identifying mutational hotspots or other defined regions in proteins (or antibodies) [220]. Such approaches allow for specific regions to be targeted, resulting in more meaningful mutations in the mutant libraries. Another approach utilizes *in vitro* scanning saturation mutagenesis to determine an antibody binding pocket for mutation [234]. In this case, an anti-digoxin scFv was determined to have six key contact residues and was targeted for mutation to study the antibody specificity and affinity towards digoxin and its analogues. This method is a systematic tool that provides a comprehensive analysis of different amino acid residues towards antibody affinity and specificity, evaluation of the plasticity of the key residues, and is suitable for preliminary structural studies for an unknown protein [234,235]. Designing an optimal degenerate primer set is the key to constructing a quality mutant library [220]. Several computational programs, including LibDesign [226], AA-Calculator [236], and DC-analyzer [220,237], have been compared, and it was concluded that DC-analyzer was able to provide the best-suited degenerate primers according to a user-defined randomization scheme [220]. The integration of computational analysis with *in vitro* methods has helped to improve the quality of mutants generated for mAb production.

### 4.3. Gene Synthesis Methods for Synthetic Antibody Gene Production

De novo gene synthesis is an ideal strategy to create desired gene sequences with the aid of high-throughput sequencing technology that offers iterative and comprehensive gene analysis. Gene synthesis is the preferred solution for gene diversification in several instances. It is well-suited for modifications to gene sequences and allows for specific design of DNA constructs. The strategy is particularly helpful to study the influence of modified sequences on particular functions of recombinant antibodies for improving the phenotypic features of antibodies. In addition, natural constructs are sometimes inaccessible, while gene synthesis provides a direct solution to obtain target sequences [238,239]. Conventional gene synthesis is driven by oligonucleotide synthesis employing solid-phase phosphoramidite chemistry. The underlying principle of phosphoramidite-based oligonucleotide synthesis consists of four main steps (deprotection, coupling, capping, and oxidation) to insert one base at a time to a growing oligonucleotide chain fixed to a solid support [240]. Generally, the synthesis takes place in small individual columns and the oligonucleotides are subjected to purification prior to quality assessment. The automated process can cater to the synthesis of a large number of oligonucleotides in parallel (up to 100 nmol) with low error rates (one base error in 200 nucleotides) [238,241].

High-throughput synthesis of oligonucleotides is now available on an array-based oligonucleotide synthesis platform. The initial Affymetrix method had limited flexibility, in which each array model requires customized photolithographic masks. The mask is important to directing UV light over the solid substrate during each synthesis process. It selectively deprotects and activates the 5′-hydroxyl group in the growing chain to allow for the incorporation of free nucleotides [239,242]. To date, several technologies have been introduced that do not require the masking technique. Inkjet-based printing was proposed by Blanchard et al. and merged with standard oligonucleotide synthesis chemistry to produce oligonucleotide arrays [243]. Briefly, the inkjets can emit picolitres of free nucleotides onto a chemically modified glass slide, allowing the coupling of phosphoramidites for DNA synthesis. The defined area is designed to contain the droplets to avoid mixing with adjacent drops, offering precise and high-density DNA synthesis. NimbleGen Systems uses a programmed, automated micromirror device to direct light at specific sites on an array (up to pixel scale) without masking. This technology offers customization of small volumes of an array at a lower cost than the cost of a photolithographic mask (which are cheaper for large-scale manufacturing) [161,244]. This array has enabled the fabrication of a complete human protein peptide array for epitope mapping of antibodies [245]. CustomArray (CombiMatrix) utilizes a modified semiconductor that contains arrays of platinum microelectrodes that allow for digital control of oligonucleotide synthesis. The microelectrodes can be activated by electrochemical reactions to generate acid for deprotection of the growing nucleotide chain [246]. The array also allows for the fixation of biomolecules, such as antibodies. Antibodies are fixed onto the array microchip’s surface and the array can be subsequently used for immunoassays. The array platform offers high sensitivity and specificity at a multiplex level [247,248]. All the above platforms generate DNA arrays according to a customizable configuration. The common advantage for all the technologies is array miniaturization; therefore, only a small amount of reagents is needed for an array [238]. Nevertheless, array-based synthesis, such as NimbleGen and CustomArray, exhibits high error rates during simultaneous production of longer and multiple oligonucleotides. The resulting errors, such as undesired substitutions and indels, can be attributed to problems with depurination and nucleotide incorporations, thereby greatly affecting the overall product quality. Purification steps are subjected to the synthesis product for removal of erroneous sequences, including high performance liquid chromatography (HPLC) and polyacrylamide gel electrophoresis (PAGE) [249].

It is undeniable that the challenges of array-based synthesis are mainly associated with its miniaturization, in which the concentration of individual oligonucleotides on an array may be insufficient for priming and result in higher error rates of the resulting oligonucleotide pool [239]. However, there are some examples of this issue being successfully overcome and that demonstrate the array-based strategy for DNA synthesis [250,251]. A programmable DNA microchip was employed and divided into sections. Initially, an array of oligonucleotides was generated, followed by amplification on one area of the microchip. Next, hybridization of the synthesized cleaved oligonucleotides to complementary oligonucleotides that spanned to the second area was performed to detect any sequence errors. The error-free fragments were then assembled into full-length DNA fragments. Nonetheless, assembly of a large pool of oligonucleotides employing this method is impractical as cross-hybridization may arise given the huge diversity of the pool [238]. Alternatively, selective oligonucleotide pool amplification directed by predetermined barcoding is useful for assembling specific gene fragments into full constructs. Each barcode represents an individual gene fragment and is digested prior to gene assembly [252]. Recently, few scFv gene libraries have been constructed using this approach via parallel array synthesis of degenerate oligonucleotides on two DNA microchips [253]. A humanized anti-ErbB2 antibody (HuA21) was diversified at its CDR through small perturbation mutagenesis and post-validation using deep sequencing prior to mutant screening by phage display. The method involves DNA synthesis on small polystyrene beads and depositing a mixture of those beads on a fibre optic array, resulting in randomly assembled DNA arrays. In the earlier versions of the arrays, the beads were encoded with different combinations of fluorophores, allowing for the oligonucleotides on the array to be optically distinguished at precise positions, termed ‘decoding the array’ [254]. The fluorophore encoding method was superseded by the current decoding methods, which involve the hybridization of short, fluorescent labelled oligonucleotides in a sequential manner. The improved methods cater to the use of a large number of beads on an array, and the functionality of the array can be validated prior to use [255].

Slonomics^®^ technology, in contrast to the conventional gene synthesis approach, completely eliminates the dependency on single-stranded oligonucleotides synthesis and is well-adapted for a fully automated gene synthesis system [256]. The method involves a library of standardized, chemically synthesized DNA building blocks and a series of repeating reaction steps (pipetting, mixing, incubation, and washing) for controlled synthesis of a highly diverse mutant gene library. The DNA standard building blocks are single-stranded oligonucleotides with self-complementary regions, forming a stable hairpin-like secondary structure with a three-nucleotide single-strand overhang to allow ligation with another building block. To produce larger dsDNA constructs, the scaffold that is made up of two building blocks is enzymatically cleaved to allow for the incorporation of the three-base single-stranded overhangs to the growing chain, and the reaction cycle is repeated five times. The codon triplets can be designed to encode for all 20 amino acids, which enables the generation of variants. SlonoMax libraries are produced using this proprietary technology [257]. A synthetic Fab library was constructed with a natural mimic design using Slonomics^®^ technology that offers precise control over amino acid frequencies in the created diversity [224].

gBlocks^®^ gene fragments (Integrated DNA Technologies, IDT) are short-to-medium-length DNA fragments that are synthesized to consist of the desired gene modifications and are readily used in gene cloning. These dsDNA blocks are synthesized under controlled conditions, and the core of their application is gene construction and editing. Gene fragment libraries are a mixed pool of DNA fragments comprising of 18 consecutive N bases or K (where K = G/T) bases that are used to synthesize gBlocks. Upon synthesis, the products are subjected to quality control assessments, including capillary electrophoresis (to check for fragment length) and mass spectrometry (to check for sequence composition). For gene editing purposes, gBlocks are used to modify the target region by introducing indels. Specific primers are designed to prime the target region for editing and subsequent removal of the target region, which can be replaced by gBlocks [249]. Hence, gBlocks synthesis has allowed the synthetic generation of antibody libraries with desired modifications [258] as well as the complete synthesis of monoclonal antibody constructs [259].

The development of oligonucleotides and gene synthesis technology also leverages the introduction of Next Generation Sequencing (NGS) technologies [46]. NGS technologies are used for screening and selecting the best sequences prior to assembly. The rationale for using such an approach is to reduce any possible errors and ultimately enhance either the quality of the generated DNA library or the synthetic constructs [238]. Such an error-correction approach is particularly interesting and has been applied during the development of DNA and antibody libraries [260,261,262]. In terms of antibody discovery, NGS has been a major advantage in streamlining the antibody discovery and development pipeline. NGS provides all the essential sequence information of the antibody repertoire in an antibody library [263] and can be coupled with antibody phage display panning to select antibodies against targets [264]. The affinity-selected polyclones after panning rounds can be deciphered individually based on sequence variability and followed by in-depth analysis of the affinity-selected pool. NGS panning can only provide sequence information, and the correlation to the binding affinity of antibodies is still required. Conventional immunoassays are done to confirm the binding ability and specificity towards the targets [264]. The in-depth analysis of the antibody pool would be meaningful to provide insights; for instance, the frequency of occurrence by each V-gene family that may have a correlation to the antigen–antibody binding preference [265]. Kono et al. used NGS analysis to compare the mice naïve repertoire with respect to the mice antigen-specific repertoire and revealed a complete mice B-cell repertoire landscape [266]. A series of bioinformatics tools are available to aid in deciphering the NGS data [258,265,267,268]. Taken together, the advancements in molecular and gene synthesis technologies will help to improve the refinement processes of recombinant antibodies, enabling mAbs to play a bigger role in biomedical applications. A summary of the available mutagenic libraries generated for antibody-related studies is provided in Table 2. 

## 5. Conclusions

There are several considerations that should be considered before randomization of CDR is carried out to avoid unwanted effects to the parent clone. Random degeneracy of CDR using degenerate primers may cause some issues, such as misfolding, a low expression level, toxicity to *E. coli* hosts, and genetic instability. Improved strategies have been applied such that fine tuning is performed solely on the canonical structure of the CDR and amino acids that are involved in antigen binding or grafting CDR to single or multiple frameworks to display similar natural diversities in the immune system. The focused CDR randomization is able to control and minimize variations of the CDR canonical structures, allowing for proper folding and improved expression of functional frameworks. The sequence diversity of antibody CDR or framework regions has tremendously contributed to the affinities of the variants residing in the antibody repertoires, whether it is a natural or synthetically made repertoire. The continuous effort to develop various molecular strategies to improve antibodies, especially for therapeutic and diagnostic candidates, is mainly imposed on CDR and framework regions. Given that it is impractical to generate all possible combinations of residues residing in the CDR regions, focused mutagenesis is applied to selected regions, such as antibody regions in contact with the antigen (CDR loops) or the framework regions that have a great influence on stability, solubility, expression levels, and affinity of the antibody. The framework regions are generally situated in close proximity to the CDR and have been known to also contribute to antigen binding. Although there are many strategies available to perform either random or focused mutagenesis, the key to obtaining desired variants is to impose the correct mutagenesis onto precise residue(s) or regions that are located on the paratope. In addition, different combinations of mutations will impact the resulting variant on various levels. All of these are extremely complicated; therefore, *in silico* analysis and prediction methods are helpful to obtain intrinsic information and enable rational considerations to be made prior to experiments. On top of that, the generation of variants can be rapidly validated by high-throughput DNA sequencing for sequence diversity analysis and proceed to direct screening in parallel. At present, the available molecular-based strategies are developing at a rapid pace, which can reduce the development timeframe as well as cost for affinity maturation. Finally, the constant advancement of *in silico* strategies, machine learning algorithms, and automation have impacted the way current antibody discovery and design is being carried out (for a review, see Roy et al. [229]). The convergence of new molecular strategies coupled with novel high-throughput sequencing methods and computational analysis will undoubtedly aid in the rapid progress of recombinant mAb development and engineering.

## Figures and Tables

**Table 1:** Various phage display antibody libraries and their attributes.

Library	Library Name/Author	Format	Source/Diversity	Library Size	Targets	Affinities	Reference
Naïve	Marks et al.	scFv	human	1.9 × 10^8^	Lysozyme, Haptens	nM	[66]
	CAT 1.0 MedImmune	scFv	human	1.4 × 10^10^	Fluroscein, Hapten, hormones	nM	[65]
	CAT 2.0 MedImmune	scFv	human	1.29 × 10^11^	Peptides, Receptors, Chemokines, Cytokines, Growth factors, Protease inhibitor, IgE, gp41	nM–pM	[102]
	de Haard et al.	Fab	human	3.7 × 10^10^	TTX, phOx, MUC1, human glycoprotein hormones	nM	[103]
	Omar et al.	Fab	human	2.99 × 10^9^	LF recombinant *Bm*SXP antigen	ND	[104]
	Lim et al.	scFv	human	2 × 10^9^	HlyE	ND	[105]
	Li et al.	scFv	human	9 × 10^9^	Human N-cadherin	nM	[106]
	Kim et al.	Fab	human	3 × 10^10^	Human recombinant proteins, peptides	nM	[107]
Immune	Huse et al.	Fab	murine	2.5 × 10^7^	NPN	nM	[108]
	Kramer et al.	scFv	human	9.3 × 10^6^, 1.2 × 10^7^	Rabies virus (RV) glycoproteins (gp)	ND	[109]
	Burton et al.	Fab	human	10^7^	HIV-I surface glycoprotein gp120	nM	[110]
	Hamidon et al.	scFv	human	10^9^	Recombinant MTb α-crystalline	ND	[111]
	Rahumatullah et al.	scFv	human	10^8^	LF recombinant *Bm*SXP antigen	ND	[112]
Semi-synthetic	Hoogenboom and Winter	scFv	H, 49 V_H_/1 V_L_	2.2 × 10^7^	Haptens, TNF	ND	[85]
	de Kruif et al.	scFv	H, 49 V_H_/7 V_L_	3.6 × 10^8^	DNP, TTX, GBS, SpA, HMG, IgG, Tg, VWF, A2, ICAM-1, δEGP-2, BLT1, PBX1a	μM–nM	[113]
	Tomlinson I+J	scFv	H, 1 V_H_/1 V_κ_	1.47 × 10^8^*	Human fibrin clots	ND	[114]
	Nissim et al.	scFv	H, 50 V_H_/1 V_L_	1 × 10^8^	FITC, NIP, phOX, KLH, maltose BP, TCR, BiP, EF-1α, SRY, anti-erythrocyte rhesus D antibody, p53	ND	[95]
	Pini et al.	scFv	H, 1 V_H_/1 V_κ_	3 × 10^8^	Recombinant fibronectin fragments	nM–pM	[92]
	Hairul et al.	sdAb	H, 1 V_H_	6.6 × 10^9^	Recombinant MTb α-crystalline	ND	[115]
	n-CoDeR^®^	scFv	H, 1 V_H_/1 V_L_	2 × 10^9^	Haptens, peptides, carbohydrates, proteins	nM	[83]
	Dyax	Fab	H, 1 V_H_/1 V_L_	4.5 × 10^10^	TIE-1, DESC1, MSPL, hK1	nM	[84]
	Chen et al.	sdAb	H, 1 V_H_	2.5 × 10^10^	Vaccinia protein B5R	nM	[88]
	Lee et al.	Fab	1 V_H_/1 V_L_	4 × 10^10^	mVEGF	nM	[116]
	Griffiths et al.	Fab	49 V_H_/26 V_κ_/21 V_λ_	6.5 × 10^10^	NIP, FTIC	μM–nM	[68]
Synthetic	Ylanthia	Fab	36 V_H_/V_L_ pairing	1.3 × 10^11^	rhTNF-α, M-CSF, rhErbB4, rhFZD-4, eGFP	nM–pM	[94]
	HuCAL^®^	scFv	49 V_H_/V_L_ pairing	2 × 10^9^	ICAM-1, Insulin, CD11b, hEGFR, Mac1p, Hagp, NFκBp	nM–pM	[99]
	HuCAL^®^	Fab	49 V_H_/V_L_ pairing	2.1 × 10^10^	rFGFR3	nM	[117]
	HuCAL GOLD^®^	Fab	49 V_H_/V_L_ pairing	1.6 × 10^10^	IL18R-Fc, β-Gal, Est-BSA	pM	[69]
	HuCAL PLATINUM^®^	Fab	49 V_H_/V_L_ pairing	4.5 × 10^10^	Receptor, interleukin, virus, growth factor, peptide, cytokine, IgG1(1), IgG1(2)	nM–pM	[118]
	ETH-2-Gold	scFv	2 V_H_/V_L_ pairing	3 × 10^9^	BSA, TNC, TTX, haptoglobin, hemoglobin, HCV envelope proteins, fibronectins	nM	[86]
	PHILO	scFv	2 V_H_/V_L_ pairing	3.1 × 10^9^	Fibronectin domains, murine tenascin-C domain, fibrin	nM	[90]
	PHILODiamond	scFv	2 V_H_/V_L_ pairing	ND	Fibronectin, TNC, fibrinogen, GST, MMP1, MMP3, mycolactone, collagen I, follistatin-like protein I, PSMD6, serpin, TIMP, UBOL1, TOM	nM	[119]

scFv: Single chain variable fragment; Fab: Antigen-binding fragment; sdAb: Single domain antibody; H: Human; ND: Not determined; NPN: KLH coupled p-nitrophenyl phosphonamidate antigen I; TNF: Tumour necrosis factor; DNP: Dinitrophenyl; TTX: Tetanus toxoid; GBS: Group B streptococcal type III capsular polysaccharide; SpA: Human surfactant protein; HMG: HMG box domain of T-cell specific transcription factor; Tg: thyroglobulin; VWF: Von Willebrand factor; A2: VW fragment 2; ICAM-1: intracellular adhesion molecule I; δEGP-2: deletion mutant of the epithelial glycoprotein EGP-2; BLT-1: Brain, lung T-cell specific DNA binding protein; PBX1a: Pre-B homebox 1a; FITC: Fluorescein isothiocyanate, NIP: 4-hydroxy-5-iodo-3-nitrophenylacetyl; phOx: 2-phenyl-5-oxazolone; KLH: Haemocyanin from keyhole limpet; maltose BP: Maltose binding protein; TCR: Soluble chimeric murine T-cell receptor; BiP: Recombinant rat immunoglobulin binding protein; EF-1α: Human elongation factor 1α; SRY: Human sex-determining region Y protein; p53: Human tumour suppressor protein p53; MUC1: Breast cancer associated antigen; HIV-I: Human immunodeficiency type I; LF: Lymphatic filariasis; MTb: *Mycobacterium tuberculosis*; HlyE: *Salmonella typhi* hemolysin E; mVEGF: Murine vascular endothelial growth factor; TIE-1: Tyrosine kinase with Immunoglobulin like And EGF like domains 1; DESC1: Endotheliase 1; MSPL: Endotheliase 2; hK1: Human tissue kallikrein; rhTNF-α: Recombinant human tumour necrosis factor alpha, M-CSF: Macrophage colony-stimulating factor, rhErbB4: Recombinant human tyrosine kinase receptor 4; rhFZD-4: Recombinant human frizzled class receptor 4; eGFP: Enhanced green fluorescent protein; CD11b: Type 1 transmembrane glycoprotein (Mac-1); hEGFR: Human epidermal growth factor receptor; Mac1p: Mac-1 peptide; Hagp: Hag peptide; NFκBp: NFκB peptide; rFGFR3: Recombinant human fibroblast growth receptor 3; IL18R-Fc: Fc- conjugated interleukin-18 receptor alpha; β-Gal: β-galactosidase; Est-BSA: β-estradiol coupled to bovine serum albumin; TNC: C-domain of tenascin-C; HCV: Hepatitis C Virus; GST: Glutathione-S-transferase; MMP1/3: Human matrix metalloproteinase 1/3, PSMD6: Proteosome 265; TIMP: Tissue inhibitor of metalloproteinase; UBQL1: Ubiquilin I; TOM: Translocase of the outer membrane. *Obtained from manufacturer’s protocol.

**Table 2:** Antibody-related mutagenic libraries generated via *in vitro* affinity maturation methods.

Methods	Description	Mutational Rate *			Library Size/Diversity	Affinities	Affinity Selection	Ref.
		High	Moderate	Low				
Error-prone PCR	Randomization of an scFv (digoxin/digoxigenin)	+	+	+	10^5^–10^6^	nM	FACS and SPR against 100 nM digoxin	[158]
	Randomization of V_H_/V_L_ of 3 Fab (progesterone)	+	+		ND	µM–nM	ND	[129]
	Randomization of several monobodies (fibronectin type 3)			+	10^7^–10^9^	nM	Lowering antigen (MAP2K5 and SF3A1) concentrations sequentially from 300 nM to 10 nM	[139]
	Randomization of CDR1 and CDR2 of an scFv (CEA)		+	+	10^7^	nM	ND	[269]
	Randomization of a Fab (streptavidin)	+		+	10^6^	nM	Lowering antigen concentrations sequentially from 5% to 0.5% (initial is 6 nM)	[144]
	Generation of a hemagglutinin mutant library	ND			ND	ND	Selected vaccine candidates were evaluated on mice protection study	[131]
	Using spiked genes for random mutations	+			ND	ND	ND	[132]
	Generation of hyperdiversified human antibody fragment mutant libraries using MutaGen™		+	+	10^6^–10^7^	ND	ND	[134]
	Generation of scFv gene mutant library using RCA		+	+	10^7^	ND	ND	[135]
	Randomization of an scFv (NP)		+		ND	nM	Gradually decreasing antigen concentration from 8 nM to 1 nM and repeat two rounds with 1 nM	[137]
	Randomization of an scFv (fluorescein)	ND			10^5^–10^7^	fM	Competitive panning against fluorescein competitor and FACS	[138]
Chain recombination	Light chains shuffling (V_ĸ_ and V_λ_) of an scFv (phOx-15)		+	+	10^6^	nM	Two rounds of panning against 10 µg/mL antigen	[150]
	V_H_ chain shuffling of an scFv (phOx-15)		+		10^5^	nM	Four rounds of panning against 1 µg/mL antigen	
	V_H_/V_L_ shuffling of several Fab (NPN)	ND			10^6^	ND	ND	[148]
	V_H_/V_L_ shuffling of several scFv (s-triazine) #coupled with random point mutations	ND			10^6^–10^7^	nM	Three repetitive cycles using immunoaffinity chromatography	[149]
	V_H_/V_L_ shuffling of an scFv (c-erbB-2)		ND		10^6^	nM	Gradually lowering antigen concentration from 100 nM to 1 nM vs. 40 nM to 0.01 nM	[151]

	V_H_/V_L_ shuffling of chimeric antibodies (Lewis Y)	ND			ND	ND	SPR	[152]
	V_L_ shuffling of a Fab (KDR)	ND			10^8^	nM	Gradually decreasing phage input and time for binding	[153]
Site-specific mutagenesis	Saturation mutagenesis of an scFv (progesterone) #coupled with random mutagenesis	ND			10^6^	nM	Five rounds of competitive selection against 5 nM antigen and 5 µM competitor	[164]
	Kunkel mutagenesis and asymmetric PCR on FN3 monobodies	+			10^8^	2 to 4-fold higher	Gradually decreasing antigen concentration from 25 nM to 500 pM with increasing washes	[175]
	Defined positions in the CDR to construct four Fab (VEFG)	ND			ND	nM	Competitive phage ELISA with as low as 100 nM antigen	[176]
	Overlap-extension mutagenesis and microarray-based DNA synthesis of p53 and Gal4	+	+		ND	NC	NC	[185]
	DNA shuffling using ssDNA and lambda exonuclease #coupled with CDR3 mutagenesis using NNK nucleotides	ND			10^7^–10^8^	NC	NC	[186]
	Chain recombination via specific DNA hybridization on an scFv	NC			ND	NC	NC	[190]
	Mutational hotspot mutagenesis on CDR2/3 of a peptide (VHH)	ND			10^11^	nM	Three rounds of panning against 100 nM antigen	[194]
	*In vitro* somatic hypermutation with AID of humanized antibodies	ND			ND	pM	SPR analysis	[197]
	Germline hotspot mutagenesis of an antibody, RFB4	NC			ND	ND	Subtractive biopanning with increasing washes	[198]
	Single and multiple mutations on CDR2/3 of a nanobody (α-synuclein)	ND			10^6^	nM	FACS against decreasing peptide concentration (from 50 nM to 5 nM)	[202]
	Randomization of CDR3 of a V_H_/ VHH domain with controlled codon ratios using ProxiMAX strategy	ND			ND	ND	NC	[224]
	Selected V_H_/V_L_ framework pairs were randomly combined for constructing mutant Fab Ylanthia libraries	ND			10^11^	nM–pM	Few human recombinant antigens were used.	[94]
	Single-site saturation mutagenesis was performed using PFunkel mutagenesis for TNF, pertussis toxin, and TROP2 mutant libraries	ND			ND	ND	Selection against 32 nM scFv (TNF), 5 nM anti-pertussis, 22 nM Fab (TROP2) using FACS	[216]
	Affibody (Aβ) mutant libraries construction using SlonoMax^®^	ND			10^7^–10^8^	nM–pM	Gradually decreasing antigen concentration from 50 nM to 10 nM using FACS	[257]
Gene synthesis	Five scFv (erbB2) gene libraries were constructed on an array	ND			10^6^	рM	Gradually decreasing antigen concentration from 0.1 nM to 0.001 nM	[253]
	Sequence-defined oligonucleotide libraries were assembled on a microarray and combined into an scFv mutant library	ND			ND	ND	Four rounds of selection on PVRL4	[260]
	Random V_H_ and V_L_ paired libraries generation using OE-RT-PCR in microemulsion	ND			ND	nM	9 mM of Human IgG1-Fc and 9 mM of IL-21 proteins were used in FACS	[259]

PCR: Polymerase chain reaction; scFv: Single-chain variable fragment; FACS: Fluorescence-activated cell soring; SPR: Surface plasmon resonance; Fab: Antigen-binding fragment; ND: Not determined; MAP2K5: mitogen-activated protein kinase kinase 5; SF3A1: Splicing factor 3A subunit 1; CDR: Complementarity determining region; CEA: Carcinoembryonic antigen; RCA: Rolling circle amplification; NP: 4-hydroxy-3-nitrophenyl acetyl; phOx: 2-phenyloxazolone; NPN: Nitrophenyl phosphonamidite; c-erbB-2: Glycoprotein tumour antigen; Lewis Y: Carbohydrate epitope; KDR: Kinase inserting domain-containing receptor; NC: Not applicable; VEGF: Vascular epithelial growth factor; p53: Human tumour suppressor; Gal4: Yeast transcription factor; VHH: Heavy chain variable domain antibody or nanobody; RFB4: Anti-CD22 monoclonal antibody; SDR: Specificity determining residues; TNF: Tumour necrosis factor; TROP2: Tumour-associated calcium signal transducer 2; Aβ: Amyloid beta; PVRL4: Poliovirus receptor-related 4; OE-RT-PCR: Overlapping extension real-time polymerase chain reaction. * Mutational rate ranking is an approximation according to the listed articles. Many articles do not determine mutational rates.

## Abbreviations

mAb	Monoclonal antibody
CDR	Complementarity determining regions
SHM	Somatic hypermutation
scFv	Single chain variable fragment
Fab	Antigen binding fragment
Fv	Variable fragment
GlySer	Glycine-serine linker
ssDNA	Single-stranded deoxyribonucleic acid
HC	Heavy chain
LC	Light chain
PCR	Polymerase chain reaction
RCA	Rolling circle amplification
dNTPs	Deoxyribonucleoside triphosphates
SM	Saturation mutagenesis
TAG	Amber stop codon
dsDNA	Double-stranded deoxyribonucleic acid
OE-PCR	Overlap extension polymerase chain reaction
sdAbs	Single domain antibodies
ISM	Iterative saturation mutagenesis
B-FIT	B-factor iterative test
TNF	Tumour necrosis factor
UV	Ultraviolet
HPLC	High performance liquid chromatography
PAGE	Polyacrylamide gel electrophoresis
NGS	Next generation sequencing
